# Hemodynamic homeostasis disequilibrium in critical illness

**DOI:** 10.3389/fphys.2025.1503320

**Published:** 2025-07-15

**Authors:** Jie Wang, Xiaoting Wang, Dawei Liu, Hui Lian, Guangjian Wang, Zewen Tong, Qingyu Deng, Qirui Guo, Qian Zhang, Yangong Chao, Wanhong Yin

**Affiliations:** ^1^ Department of Critical Care Medicine, Peking Union Medical College Hospital, Peking Union Medical College and Chinese Academy of Medical Sciences, Beijing, China; ^2^ Department of Health Care, Peking Union Medical College Hospital, Peking Union Medical College, Chinese Academy of Medical Sciences, Beijing, China; ^3^ Department of Critical Care Medicine, First Hospital of Tsinghua University, Beijing, China; ^4^ Visualized Diagnostics and Therapeutics & Artificial Intelligence Laboratory/ Department of Critical Care Medicine, West China Hospital, Sichuan University, Chengdu, China

**Keywords:** hemodynamic homeostasis, hemodynamic homeostasis disequilibrium, hypoxia, critical unit, HOUR (Host/Organ Unregulated Response)

## Abstract

Over millions of years, the circulatory system evolved from primitive forms into a highly specialized network capable of overcoming time-distance constraints and enhancing diffusion efficiency. This structural advancement laid the physiological foundation for the regulation of hemodynamics and systemic homeostasis. Hemodynamic homeostasis is a fundamental biological process that ensures the continuous delivery of oxygen and substrates while facilitating the removal of carbon dioxide and metabolic waste. Such balance is essential for sustaining cellular metabolism and maintaining the function of vital organs throughout embryonic development and the human lifespan. Disruption of this equilibrium, primarily driven by the Host/Organ Unregulated Response (HOUR), compromises the cardiovascular-respiratory system, resulting in hemodynamic homeostasis disequilibrium. HOUR specifically targets the *critical unit*—a constellation of elements essential for oxygenation and cell energetics, including the microcirculation, endothelial glycocalyx, and mitochondria, impairing the oxygenation process, ultimately triggering critical illness. Although intervention targeting systemic hemodynamic variables (e.g., pressure, flow) may temporarily improve regional perfusion, restoring full homeostasis remains challenging. This is largely due to the activation of multiple positive feedback loops (e.g., coagulation cascades) and impairment of key negative feedback mechanisms (e.g., blood pressure regulation). In the presence of ongoing HOUR, inappropriate or delayed interventions may exacerbate injury and accelerate irreversible organ damage or death. Therefore, it is both essential and urgent to elucidate the initiation, recognition, progression, and modulation of hemodynamic homeostasis disequilibrium.

## Highlights

1. Hemodynamic homeostasis maintains systemic oxygen balance through coordinated interaction between the cardiovascular and respiratory systems, with rapid reflex regulation mediated by neural and endocrine mechanisms.
2. Reflex circuits and feedback loops respond to physiological insults by activating sensors, controllers, and effectors. Negative feedback loops preserve homeostasis, whereas positive feedback loops exacerbate injury and drive hemodynamic homeostasis disequilibrium.
3. HOUR encompasses dysregulated neural, neuroendocrine, inflammatory, immune, coagulative, bioenergetic, and metabolic responses that impair the oxygenation process, initiating a cascade that leads to hemodynamic homeostasis disequilibrium and critical illness.
4. Hemodynamic homeostasis disequilibrium is characterized by anatomic mismatch and disordered feedback, reflecting impaired integration between cardiovascular-respiratory system and neural reflex pathways.
5. HOUR-mediated injury to critical units-including endothelial glycocalyx and mitochondria-leads to dysoxia and the activation of self-sustaining positive feedback loops, distinguishing this process from classical immune-mediated pathology.

## Introduction

Shock, hemodynamic compromise, and hemodynamic instability-often manifesting as hypotension, hypertension, tachycardia or arrhythmias, hyperlacticaemia, or even cardiac arrest-are the commonly used terms to describe a host in a critically unstable hemodynamic state (Vincent and De Backer, 2013; Goldstein et al., 1990; Vieillard-Baron et al., 2003). However, these conditions represent advanced stages of hemodynamic homeostasis disequilibrium, that can easily precede sudden deterioration or death. Typically, clinical hemodynamic monitoring and interventions are only initiated once such overt manifestations are present (Cecconi et al., 2014; Rhodes et al., 2017).

**FIGURE 1:**
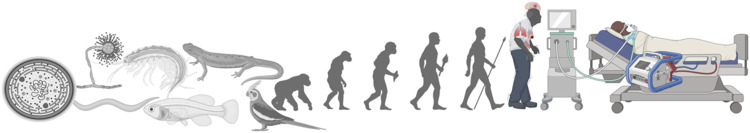
Evolutionary continuum from single-cell organisms to modern critical illness. This illustration depicts the evolutionary trajectory of biological complexity—from unicellular organisms to vertebrates and eventually Homo sapiens—highlighting the emergence of integrated cardiovascular-respiratory systems. The rightmost progression shows the modern critically ill patient supported by advanced life-sustaining technologies, such as mechanical ventilation and extracorporeal membrane oxygenation (ECMO). This continuum emphasizes how evolutionary adaptations for oxygen regulation and energy balance are challenged under conditions of critical illness.

Hemodynamic homeostasis is a product of long-term biological evolution. As illustrated in Figure 1, the evolutionary transition from unicellular organisms to complex vertebrates—including humans—has enabled the development of an integrated cardiovascular-respiratory system to maintain oxygen delivery and energy balance. Under physiological conditions, blood circulates from the heart through arteries, arterioles, capillaries, venules, and veins, returning to the right heart, forming a continuous and coordinated flow loop. To better understand and quantify dynamic and energy-efficient regulation between the cardiovascular and respiratory systems that sustains this circulation, mathematical modeling grounded in variational principles—such as the Euler-Lagrange formulation—offers a powerful framework for simulating these complex interactions. By integrating both systems into a unified cardiovascular-respiratory system (CVRS) model, this framework enables the optimization of time-dependent workloads associated with oxygen transport and blood flow delivery (Calderon et al., 2017). This systemic optimization underscores the importance of each step in the oxygen cascade, from environmental intake to cellular utilization.

While systemic circulation and oxygen delivery can be optimized through cardiovascular-respiratory integration, the ultimate success of oxygen transport depends on efficient microcirculatory function and cellular utilization. Hemodynamic coherence—the alignment between macrocirculatory and microcirculatory flow-ensures adequate tissue perfusion, reflecting the integrated interplay between metabolic demand and cardiovascular-respiratory adaptations (Ince, 2015). Capillaries, primarily composed of endothelial cells, are the functional units of microcirculation and represent the principal sites of substrate and gas exchange, such as pulmonary oxygenation. To maintain oxygen supply at the cellular level, oxygen traverses a progressive pressure gradient along the oxygen cascade, from ambient air to the mitochondrial matrix (Ortiz-Prado et al., 2019). Mitochondria, the final destination of oxygen, require a partial pressure of approximately 1–10 mmHg to sustain aerobic metabolism and preserve cellular homeostasis (Zoneff et al., 2024).

**FIGURE 2:**
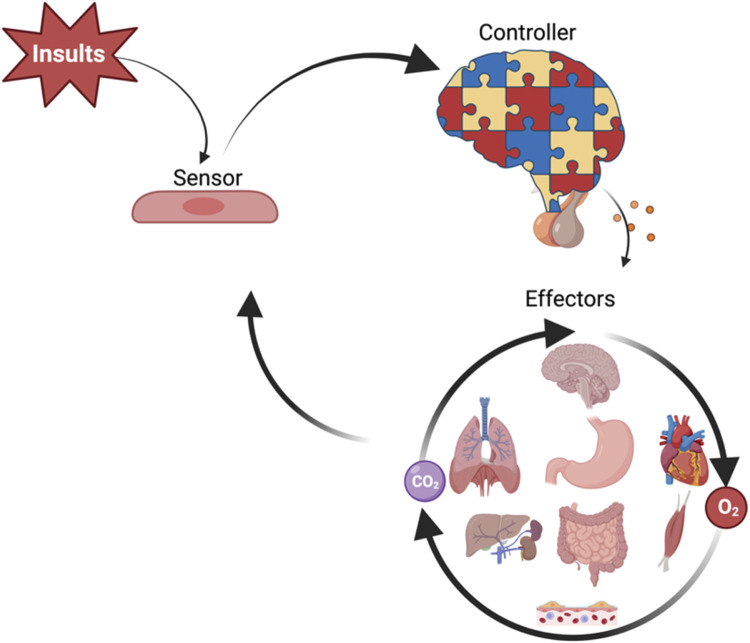
Schematic overview of hemodynamic homeostasis regulation. Systemic insults are sensed by peripheral cells (sensors), which relay signals to central controllers—primarily neural and neuroendocrine centers—that integrate input and generate regulatory outputs. These signals target systemic effectors, including the heart, lungs, kidneys, gastrointestinal tract, liver, skeletal muscle, and microvasculature, to maintain oxygen delivery (O_2_), carbon dioxide clearance (CO_2_), and overall hemodynamic disequilibrium. The figure illustrates the feedback loop sustaining hemodynamic homeostasis.

In health, the human body maintains hemodynamic homeostasis, characterized by stable arterial pressure, heart rate, respiratory rhythm, and oxygen gradients. As shown in Figure 2, this process is maintained through a coordinated feedback system in which peripheral sensors detect systemic changes, central controllers integrate signals, and effectors (e.g., heart, lungs, kidneys) restore equilibrium. Following critical insults—such as infection, trauma, inflammation, acute pancreatitis, traumatic brain injury, burns, or surgery—the host may lose its equilibrium and progress into critically ill. The hemodynamic response (HR) and the broader HOUR are complex biological processes that determine the trajectory toward recovery or deterioration. From an evolutionary perspective, the nervous and endocrine systems serve as the principal regulators of hemodynamic homeostasis in mammals (McEwen, 2007; Herman and Cullinan, 1997). While negative feedback loops maintain hemodynamic homeostasis, unchecked positive feedback loops may drive systemic dysregulation (Todorov and Jordan, 2002).

In light of the central role of hemodynamic homeostasis disequilibrium in critical illness, this review aims to dissect its underlying mechanisms, temporal progression, and phenotypic features, while identifying modifiable factors for targeted therapeutic interventions. To provide a structured understanding, we adopt a four-phase conceptual framework—initiation, recognition, progression, and modulation—centered on the Host/Organ Unregulated Response (HOUR). This framework emphasizes how disruptions in physiological feedback mechanisms—neural, immune, endothelial, and metabolic—drive the transition from early regulatory failure to systemic hemodynamic collapse. Through this lens, we aim to elucidate the pathophysiological evolution of this condition, highlight its phenotypic heterogeneity, and identify stage-specific targets for timely intervention.

## Definitions to clarify

Homeostasis is a self-regulating process by which an organism maintains internal stability while adapting to changing external conditions (Billman, 2020).

Allostasis refers to the process of achieving stability through change (Sterling and Eyer, 1988).

Hemodynamics encompasses fundamental parameters of cardiovascular function, such as arterial pressure and cardiac output. In the present review, “hemodynamics” refers to “the physical study of flowing blood and of all the solid structures (such as arteries) through which it flows” (Secomb, 2016).

Hemodynamic Coherence between the macrocirculation and the microcirculation is the condition in which resuscitation procedures aimed at the correction of systemic hemodynamics variables are effective in correcting regional and microcirculatory perfusion and oxygen delivery to the parenchymal cells such that the cells can perform their functional activities in support of organ function (Ince, 2015).

Critical Units include components essential for oxygenation process and cellular energetics, primarily microcirculation (capillaries, arterioles, and venules), endothelial cells (particularly the endothelial glycocalyx), and mitochondria. These elements play crucial role in perfusion, barrier integrity and energy metabolism (Wang et al., 2023).

Hemodynamic Response refers to a homeostatic process that replenishes the nutrients used by biological tissues by adjusting blood flow to areas of focal activity.

Host/Organ Unregulated Response (HOUR) denotes a maladaptive state in which persistent injury or inappropriate interventions lead to dysregulation across neural, endothelial, hormonal, bioenergetic, metabolic, and immune pathways, ultimately contributing to the development of critical illness (Huang et al., 2023).

### What is hemodynamic homeostasis?

Stefan De Hert defined it as the regulation of blood circulation to meet the demands of the different organ and tissue systems. This homeostasis involves an intimate interaction between peripheral metabolic needs, vascular adaptations to meet these needs, and cardiac adaptation to provide the driving force to circulate the blood (De Hert, 2012). More precisely, hemodynamic homeostasis refers to a dynamic regulatory process of the cardiovascular-respiratory system that ensures oxygen and substrates delivery to tissues while removing metabolic products and heat, thereby maintaining systemic homeostasis.

### What is hemodynamic homeostasis disequilibrium?

Hemodynamic homeostasis disequilibrium represents a critical pathophysiological state characterized by inadequate blood flow and oxygen delivery to cells, primarily due to HOUR-related dysregulation. This includes autonomic and neural failure, immune and coagulation dysfunction, bioenergetic and metabolic derangement, mitochondria injury, and cellular energy crisis-conditions that can culminate in irreversible organ damage or death.

## Initiation of hemodynamic homeostasis disequilibrium

Under physiological conditions, hemodynamic homeostasis is maintained by a tightly regulated network of negative feedback mechanisms, primarily involving the autonomic nervous system (ANS), baroreceptor reflex, and vascular autoregulation. The ANS modulates sympathetic and parasympathetic tone in response to sensory input from baroreceptors and chemoreceptors, thereby adjusting heart rate, vascular resistance, and cardiac contractility. The baroreceptor reflex, mediated by stretch-sensitive mechanoreceptors in the aortic arch and carotid sinus, responds rapidly to acute fluctuations in arterial pressure by altering vagal and sympathetic outflows to restore normotension. Vascular autoregulation, a local control mechanism, stabilizes tissue perfusion across varying systemic pressures, primarily via myogenic responses and metabolic vasodilators. Cardiovascular regulatory centers in the medulla oblongata integrate afferent inputs and coordinate autonomic efferent responses: sympathetic stimulation via the cardiac accelerator nerve increases heart rate and stroke volume, whereas parasympathetic output via vagus nerve reduces heart rate. In parallel, sympathetic efferents mediate vascular smooth muscle contraction within the tunica media to regulate systemic vascular resistance (SVR) (Salah et al., 2025; Carlson et al., 2008; Iordanova and Reddivari, 2025).

The initiation of hemodynamic disequilibrium is typically precipitated by acute pathological insults such as sepsis, major trauma, acute brain injury, massive hemorrhage, extensive surgery, burns, severe pancreatitis, or immune-mediated conditions (e.g., cytokine storm). These stressors activate pro-inflammatory signaling pathways, induce neuroendocrine overactivation, and initiate coagulation and complement cascades, forming interconnected positive feedback loops that amplify systemic inflammation, vascular leakage, and cardiac dysfunction (Fajgenbaum and June, 2020). At this stage, the finely tuned balance between perfusion pressure, vascular tone, and cardiac output becomes disrupted, marking the transition from adaptive allostasis to maladaptive disequilibrium.

At the molecular level, the initiation involves multiple interrelated pathophysiological pathways. Pro-inflammatory cytokines (e.g., TNF-α, IL-1β, IL-6) are upregulated through pattern recognition receptors (e.g., Toll-like receptors) in response to pathogen-associated molecular patterns (PAMPs) and/or damage-associated molecular patterns (DAMPs). These cytokines promote endothelial activation, increase vascular permeability, and depress myocardial function-forming a self-perpetuating inflammatory feedback loop (Takeuchi and Akira, 2010; van der Poll et al., 2017; Hotchkiss and Moldawer, 2014).

Concurrently, neuroendocrine stress responses are engaged through the hypothalamic-pituitary-adrenal (HPA) axis and sympathoadrenal system, resulting in elevated levels of catecholamines, cortisol, vasopressin, and renin-angiotensin-aldosterone system (RAAS) effectors (Goldstein, 2010; Chrousos, 2009). While initially adaptive, persistent or excessive activation of these pathways contributes to tachycardia, vasoconstriction, increased metabolic demand, and impaired baroreceptor sensitivity.

Furthermore, systemic inflammation triggers coagulation cascade activation via tissue factor expression and thrombin generation, often accompanied by anticoagulant depletion and fibrinolytic suppression, fostering microvascular thrombosis. This leads to impaired tissue oxygenation and cellular injury, exemplifying the positive feedback nature of hemodynamic deterioration. This shift from homeostatic negative feedback to unrestrained positive feedback loops underlies the initiation of hemodynamic homeostasis disequilibrium.

## Recognition of hemodynamic homeostasis disequilibrium

Timely recognition of hemodynamic homeostasis disequilibrium is essential for preventing progression to irreversible organ dysfunction and death. However, early detection is particularly challenging due to the complexity and heterogeneity of underlying physiological perturbations. The recognition phase relies on integrating clinical signs, laboratory markers, hemodynamic parameters that collectively indicate a breakdown in cardiovascular regulation.

Tachycardia is a known early indicator of critical illness and is included in the Modified Early Warning System (MEWS) (Mathukia et al., 2015). However, paradoxically, patients with tachycardia may have better outcomes in certain subgroups with elevated lactate levels (Na et al., 2023), highlighting the importance of contextual interpretation. Discrepancies between conventional scoring systems and emerging evidence may arise if the causal link between deviation and physiological context is overlooked. Hence, incorporating a physiologically informed interpretive framework is crucial for accurately assessing hemodynamic homeostasis disequilibrium.

The heart ensures a continuous blood flow and stable pressure through rhythmic contraction. Severe cardiac dysfunction, especially low cardiac output, disrupts this equilibrium. Critical care echocardiography enables real-time assessment of cardiac structure and function at the bedside, providing actionable insight into the etiology of shock. Initially septic cardiomyopathy was characterized by left ventricular systolic dysfunction. Later, a hyperkinetic profile-defined by a velocity time integral (VTI) > 20 cm, heart rate (HR) < 106 bpm, left ventricular area change (LVFAC) > 58%- was recognized. More recently, evidence suggests that ventricular performance in sepsis spans a spectrum from LVEF < 25% to > 70% (Dugar et al., 2023). RV dysfunction is also critical; its severity correlates with worsening LV dysfunction, and severe RV dilation may reduce LV size and performance (Hiraiwa et al., 2021). Both hypokinetic and hyperkinetic profiles, as well as right ventricular failure, are linked to poor prognosis. Furthermore, a physiological study showed that the actual ongoing effects after sepsis on autonomic and vascular failure are typical within 3 days in a resuscitated swine experiment (Carrara et al., 2022). It is not negligible that the regulatory process of hemodynamics is present during the entire course after the insults.

The vasculature transports oxygenated blood and metabolites through arteries, arterioles, capillaries, venules, and veins. In septic shock, vasoparalysis severely compromises oxygen delivery (Rhodes et al., 2017). Fundamental physical laws help explain this,

Ohm’s Law: MAP-CVP = CO*SVR.

(mean artery pressure-MAP, central venous pressure-CVP, cardiac output-CO).

Poiseuille’s Law: R∼ ΔP∼1/r^4^.

(Resistance-R, pressure drop-ΔP, radius-r).

Arterial hypotension results not only from decreased cardiac output but also from dramatically reduced SVR. Arterioles play a central role in regulating the blood pressure and flow distribution,

According to,

Definition of flow: Q = V/t.

(flow rate-Q, Volume-V, time-t).

Continuity equation: A_1_v_1_ = A_2_v_2_

(cross-sectional area-A, velocity-v).

Organs such as the brain, kidneys, and heart rely on autoregulation mechanisms to maintain constant perfusion, which collapse in advanced shock states. For example, cerebral blood flow (CBF) remains constant within a narrow MAP range but drops precipitously beyond it (Silverman and Petersen, 2024). Coronary circulatory autoregulation and renal autoregulation have also been identified (Feigl, 1989; Inscho and Lewis, 2009). Baroreceptor reflexes in the aortic arch and carotid sinus help maintain this equilibrium by modulating ANS output.

Apart from cardiac and vascular indicators, respiratory dysfunction plays a crucial role in early hemodynamic disequilibrium, particularly in critically ill patients with sepsis or ARDS. The respiratory system oxygenates the blood and expels carbon dioxide during the respiratory cycle. Signals from peripheral chemoreceptors (responding to hypercapnia, hypoxemia, acidosis, and central chemoreceptors (responding to CSF hypercapnia), as well as the lung stretch receptors modulate the respiratory drive. The control network includes the dorsal respiratory group (nucleus tractus solitarius), the ventral respiratory group (medulla), and the pontine respiratory group. Through the somatic and the autonomic nervous pathways, including the phrenic, intercostal, and sympathetic nerves, the respiratory rate and rhythm are adjusted.

Tachypnea is among the earliest signs of critical illness and is included in the Sequential Organ Failure Assessment (SOFA) score (Singer et al., 2016). It is often accompanied by increased respiratory drive and effort, which can be quantified using esophageal pressure metrics such as P 0.5 (pressure drop in the first 500 ms), and ΔPes (maximum pressure swing). These values are inversely related to lung compliance in sepsis patients (Mauri et al., 2021). A multicenter observational study showed that 15% of patients who developed acute respiratory distress syndrome (ARDS) within 7 days had a higher baseline increased driving pressure (ΔP) (Blondonnet et al., 2019). In a meta-analysis of 3,252 patients with ARDS, higher driving pressure was associated with higher mortality, and the threshold was 13-15cmH2O. Reverse triggering (respiratory entrainment), and spontaneous effort during mechanical ventilation can cause breath stacking, increased transpulmonary pressure, and patient self-inflicted lung injury (P-SILI).

**FIGURE 3:**
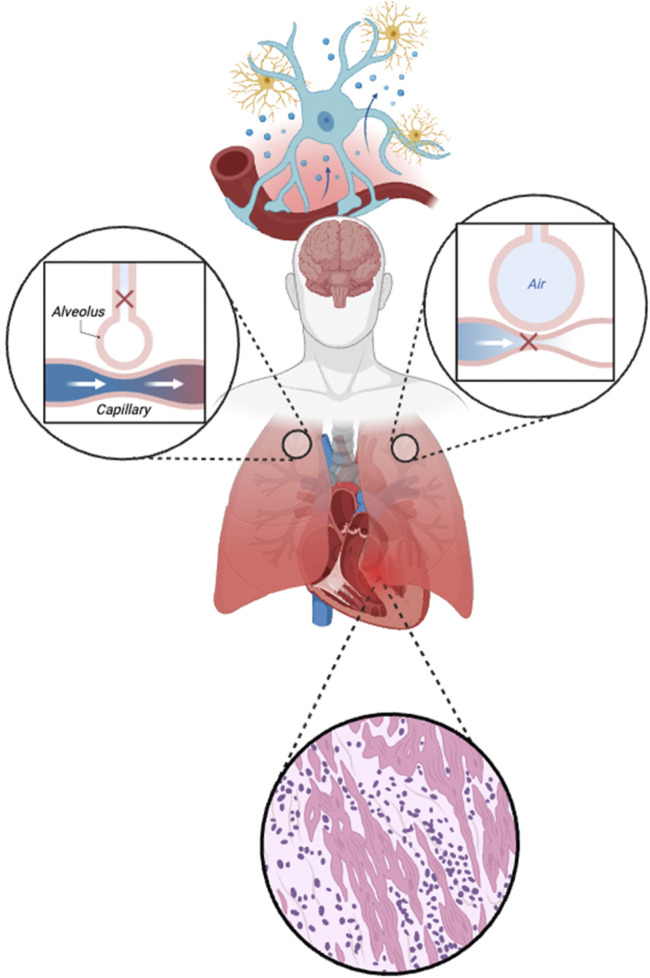
Disruption of neural control and cardiovascular-respiratory (CVRS) in critical illness. The figure illustrates damage to the regulatory brain centers (controller), pulmonary gas exchange interface, and cardiac tissue during critical illness. The top segment highlights neurovascular uncoupling and neuroinflammation, impairing autonomic and neuroendocrine control. The left and right insets show mismatched ventilation-perfusion (V/Q) relationships: impaired perfusion with preserved ventilation (dead space) and impaired ventilation with preserved perfusion (shunt), respectively. The bottom panel depicts myocardial injury characterized by inflammatory infiltration and structural disarray, further disrupting CVRS coordination and oxygen delivery.

Diaphragmatic ultrasound helps visualize and quantify changes in structure and activity. Over 50% of ventilated patients experience >10% diaphragm thickness loss, and contractile function decreases with rising driving pressure (Schepens et al., 2020; Goligher et al., 2015). In patients with ICU-acquired weakness, and diaphragm dysfunction, 2-year survival rate drops to 36%. Moreover, excessive inspiratory effort elevates transvascular pressure, contributing to pulmonary edema, reducing lung compliance, and ultimately causing ventilation heterogeneity-characterized by the pendelluft phenomenon (Enokidani et al., 2021; Chi et al., 2022). These effects can also compromise cardiac output, as the net influence of spontaneous respiratory efforts depends on intrathoracic pressure, preload, and ventricular function (Yoshida et al., 2017). Notably, RV dysfunction has been observed in up to 30% of ARDS cases (Repessé et al., 2012), further contributing to hemodynamic disequilibrium. As illustrated in Figure 3, critical illness disrupts the integrated regulation of the cardiovascular-respiratory system (CVRS), including impaired brain control centers, myocardial injury, and mismatched ventilation-perfusion coupling.

Above all, hemodynamic derangement is not an isolated phenomenon but rather a system-wide dysregulation involving dynamic interplay among cardiac, vascular, and respiratory components. This integrative disturbance, once initiated and inadequately controlled, can evolve through a cascade of molecular and systemic feedback loops.

## Progression of hemodynamic homeostasis disequilibrium

Given the heterogeneity of critical illness, recent perspectives have shifted from focusing on isolated biological alterations to recognizing interconnected clusters of dysregulation-including neural, neuroendocrine, inflammatory, immune, coagulative and metabolic pathways-as common contributors to hemodynamic homeostasis disequilibrium.

Inflammation is rapidly modulated by cholinergic signaling via the vagus, a mechanism known as inflammatory reflex (Tracey, 2002). The dysregulated immune responses in critically ill patients manifest in a biphasic trajectory-an early hyperinflammatory phase (<7 days) followed by a prolonged immunosuppressive phase (days 8 and 28). Conditions such as cytokine storm and hemophagocytic lymph histiocytosis (HLH) exemplify failure of negative feedback control (Fajgenbaum and June, 2020; Konkol and Rai, 2024). The excessive and sustained release of cytokines, interleukins, interferons, chemokines, and complements contributes to vasoplegia, arrhythmia, and myocardial dysfunction (Bozkurt et al., 2021).

In the pulmonary immune niche, interstitial macrophages (IMs) and alveolar macrophages (AMs) constitute 90%–95% of lung-resident immune cells. IMs produce high levels of IL-10, and various mediators such as nitric oxide (NO), TNF-α, and IFN-γ, while Ams-located at the mucosal interface-suppress immune overactivation and preserve pulmonary homeostasis (Kumar, 2020). Inflammatory mediators like TNF-α and IL-1 induce inducible nitric oxide synthase (iNOS) expression in immune vascular cells, linking NO generation with coagulation activation.

Inflammation and coagulation are tightly interwoven in host defense. PRRs on monocytes and derived microvesicles expose tissue factor (TF), activating the extrinsic coagulation pathway (Sriskandan and Altmann, 2008; Bach, 2006; Osterud, 1995; Pawlinski and Mackman, 2010). The TF–factor VIIa complex subsequently activates protease-activated receptors (PARs), promoting cytokine and chemokine release. DAMP stimulate platelet Toll-like receptors (TLRs) and C-type lectin-like receptors (CTLRs), which in turn activate neutrophils to generate neutrophil extracellular traps (NETs) (Foley and Conway, 2016). NETs exhibit potent platelet-binding capabilities, and contribute to microthrombus formation. Meanwhile, von Willebrand factor (vWF) enhances platelet adhesion function through GPIb. Deficiency of vWF ADAMTS13 leads to platelet thrombosis due to hyperactivated inflammation. In SARS-CoV-2 infection, pulmonary vascular endothelial injury impairs anticoagulant mechanisms. Downregulation of antithrombin and protein C pathways promotes microthrombus formation. While immunothrombosis may initially contain infection, dysregulated immunothrombosis impairs perfusion, causing tissue hypoxia, endothelial injury, and multiorgan dysfunction. NET-derived histones exacerbate epithelial permeability and thrombotic damage.

Cellular metabolic reprogramming of monocytes, neutrophils, and endothelial cells (ECs) is a critical determinant of hemodynamic control (Li et al., 2019). Anabolic and catabolic pathways influence hyperinflammation and immune tolerance. In sepsis, bacteria compete with immune cells for oxygen and glucose, while viruses upregulate glucose uptake and cellular metabolism to facilitate viral replication. The site of inflammation may be hypoxic due to thrombi and inflammatory cells. M1 and M2 macrophages metabolize glucose and free fatty acids respectively. During sepsis or inflammation, glycolytic flux leads to the stabilization of HIF-1α, which promotes activation of IL1-β in macrophages. HIF-1α links metabolic processes to immune regulation. In an *ex vivo* study, PBMCs from septic immunodepressed patients failed to produce lactate in response to LPS, demonstrating loss of Warburg Effect and dysfunction of the mTOR-HIF-1α axis (de Azambuja Rodrigues et al., 2021; Palsson-McDermott et al., 2015). IL-37, a member of the IL-1 family, exhibits protective effects by modulating cellular metabolism (Cavalli et al., 2021). Endothelial cells (ECs) in sepsis also undergo a shift from oxidative phosphorylation (OxPhos) to glycolysis, mediated by enzyme 6-phosphofructo-2-kinase/fructose-2,6-bisphosphatase isoform 3 (PFKFB3), a key glycolytic enzyme. In mouse models of LPS-induced acute lung injury, pharmacologic inhibition of PFKFB3 (e.g., 3PO) or endothelial-specific genetic deletion suppressed NF-κB signaling, downregulated ICAM-1 and VCAM-1 expression, reduced endothelial permeability, mitigated pulmonary edema, and attenuated neutrophil and macrophage infiltration (Wang et al., 2019). Knockdown of PFKFB3 in endothelial cells also inhibited nearly all TNF-α-induced pro-inflammatory factor release (Zhang et al., 2019).

Additionally, pulmonary capillaries are anatomically narrow, predisposing neutrophils to sequestration under inflammatory conditions. Adhesion and frictional forces slow perfusion, while dispersive shear forces modulate neutrophil transit (MacNee and Selby, 1993; Joffre et al., 2020). Liver dysfunction may further contribute; for example, defective hepatic triglyceride synthesis in the MAP kinase-deficient mice exacerbates endothelial damage (Frazier et al., 2009).

Altogether, immune-metabolic-coagulopathic dysregulation underlies microvascular thrombosis and endothelial dysfunction, ultimately disrupting oxygen delivery and hemodynamic coherence. These processes mark a critical inflection point in the progression toward irreversible hemodynamic homeostasis disequilibrium. As this systemic disruption deepens, cellular oxygen availability and mitochondrial function become key determinants of survival, introducing the concepts of critical unit hypoxia and mitochondrial dysregulation.

## Critical unit hypoxia

Oxygen is the fundamental substrate for mitochondrial oxidative phosphorylation, supporting adenosine triphosphate (ATP) production, maintaining redox homeostasis, regulating apoptosis, and mediating various cell signaling pathways. A deficiency in oxygen delivery (hypoxia) or impaired utilization (dysoxia) predisposes cells to bioenergetic failure. The Pasteur point represents a critical oxygen tension threshold below which aerobic metabolism becomes unsustainable, determined by the high oxygen affinity mitochondrial respiratory chain enzymes (Krebs, 1972; Rutten, 1970). Aerobic metabolism can persist at oxygen partial pressures as low as 1 mmHg (Ortiz-Prado et al., 2019).

Sustained or severe hypoxia within critical units further destabilizes hemodynamic homeostasis. Tissue oxygen desaturation was associated with a worse prognosis. For instance, irreversible cerebral injury can occur after 5–6 min of hypoxia, and myocardial necrosis may ensue after 30 min of ischemia during cardiopulmonary resuscitation (CPR). Even following the return of spontaneous circulation (ROSC), reperfusion injury contributes to oxidative stress, reactive oxygen species (ROS) generation, and metabolic dysregulation, fueling a vicious cycle of cellular damage and systemic inflammation (Adrie et al., 2004).

Lactic acidosis, a surrogate marker of impaired tissue perfusion and oxygen utilization, is strongly associated with poor prognosis and low reversibility in critically ill patients (Broder and Weil, 1964; Vincent et al., 1983; Bakker et al., 1996). In advanced stages, pulmonary manifestations-such as extrapulmonary ARDS-represent the terminal pulmonary phenotype of systemic hemodynamic homeostasis disequilibrium. Viral sepsis due to SARS-CoV-2 often demonstrates this pattern, characterized by capillary leakage, endothelial injury and severe gas exchange abnormalities (Lamers and Haagmans, 2022). Accumulated hypoxemia and hypercapnia aggravate hemodynamic homeostasis disequilibrium, perpetuating a deleterious positive feedback loop.

**FIGURE 4:**
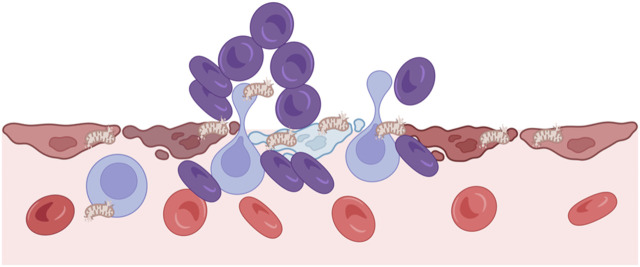
Hypoxia and *critical unit* injury. Hypoxia disrupts endothelial integrity and causes cellular damage, leading to mitochondrial release into the extracellular space. These mitochondria act as damage signals, triggering immune activation and further microvascular injury. This process contributes to local perfusion failure and systemic hemodynamic imbalance.

At the molecular level, HIF is a key transcription factor regulating cellular adaptation to low oxygen. Under hypoxia, decreased prolyl hydroxylases activity stabilizes HIF-α, enabling it to translocate into the nucleus and dimerize with HIF-β. The HIF complex binds to hypoxia-responsive elements (HREs) in gene promoters, activating downstream transcriptional programs involved in metabolic reprogramming, angiogenesis, cell survival. (Weidemann and Johnson, 2008; Taylor and Scholz, 2022). Thus, critical unit hypoxia represents a pivotal mechanism in the evolution of hemodynamic homeostasis disequilibrium. As illustrated in Figure 4, sustained hypoxia can damage endothelial integrity and trigger the release of mitochondrial contents into the extracellular space. These damage-associated signals further amplify immune activation, contributing to microvascular collapse and systemic hemodynamic imbalance.

## Mitochondria dysregulation

Mitochondria are ubiquitous in nucleated human cells, with the exception of erythrocytes, and play a central role in sustaining cellular bioenergetics and survival. The cardiovascular-respiratory system and hemodynamic response ensure adequate oxygen and nutrient delivery to support mitochondrial function after insults. However, even with seemingly preserved oxygen delivery, mitochondrial metabolism can be profoundly impaired in critical illness. Mitochondria dysfunction represents a key contributor to hemodynamic homeostasis disequilibrium, acting independently or synergistically with hypoxia (Supinski et al., 2020). During sepsis and other critical states, mitochondria undergo significant structural and functional alterations, including ROS overproduction, calcium dyshomeostasis, disruption of the electron transport chain (ETC.), impaired fusion/fission dynamics, defective mitophagy, and initiation of cell death pathways. Mitochondria-associated endoplasmic reticulum membranes (MAMs) serve as essential hubs for regulating calcium signaling, mitochondrial dynamics, mitophagy, apoptosis, and inflammatory responses (Missiroli et al., 2018). Even after resolution of the acute phase, persistent abnormalities and mitochondrial ultrastructural damage can be observed in skeletal muscle of survivors, suggesting long-lasting metabolic derangements (Klawitter et al., 2023).

## Modulation of hemodynamic homeostasis disequilibrium

Effective modulation of hemodynamic homeostasis disequilibrium requires a multi-tiered approach targeting neural circuits, central integrators, peripheral effectors, and pathophysiological mechanisms. Among these, neural regulation constitutes a rapid, pressure-sensitive mechanism that maintains perfusion stability through vasomotor tone. Neurocirculatory feedback mechanisms rely on baroreceptors and stretch receptors located in the atria, ventricles, and pulmonary tissues. For instance, reduced atrial stretch-such as during hypovolemia-triggers systemic vasoconstriction. Conversely, activation of ventricular stretch receptors promotes reflex bradycardia and vasodilation. Pulmonary congestion stimulates juxtapulmonary capillary (J receptors), inducing tachycardia, while pulmonary inflation activates mechanoreceptors leading to vasodilatory effects. (Dampney, 2016; Anand, 2019). These reflex circuits serve as protective adaptations to preserve organ perfusion after insults.

In clinical practice, interventions can be conceptualized by their modulation site within the afferent-control-effector loop. Source control measures-such as surgical drainage or antimicrobial therapy-reduce the afferent input by removing infectious or inflammatory triggers. Sedation and analgesia, targeting the central control, are widely employed to blunt exaggerated sympathetic responses. Agents like midazolam (a GABA agonist) and fentanyl (a potent opioid) are frequently used to mitigate pain, anxiety, and agitation, thereby reducing central sympathetic outflow and preventing secondary injury from overreaction. Peripheral effectors, including the heart, vasculature, lungs, kidneys, and liver are modulated via targeted supportive interventions. These include volume resuscitation, vasopressors, inotropes, oxygen therapy, renal replacement therapy (RRT), extracorporeal membrane oxygenation (ECMO), left ventricular assist devices (LVADs) and liver support systems. Together, these therapies restore systemic perfusion, oxygenation, and metabolic homeostasis, which are critical for reversing hemodynamic homeostasis disequilibrium.

A number of emerging therapies are currently under investigation for their potential to modulate hemodynamic homeostasis at molecular and cellular levels. These include: pathogen-directed agents, such as endotoxin antagonists and broad-spectrum antimicrobial peptides. Immunomodulatory therapies, including cytokine inhibitors, immune checkpoint modulators, and immunostimulants. Anti-inflammatory and antioxidant strategies: e.g., pro-resolving lipid mediators, N-acetylcysteine, vitamin C. Coagulation and purification modalities, such as anticoagulants and hemadsorption (e.g., CytoSorb), plasma exchange. Metabolic modulation, with agents like thiamine, and hydrocortisone, and targeted mitochondrial bioenergetics regulators. Endothelial protection, gut microbiome restoration, mitochondrial transplantation, and precision medicine approaches have also gained interest as systemic modulators of hemodynamic homeostasis disequilibrium (Rhodes et al., 2017; Moller et al., 2023; Borcherding and Brestoff, 2023). While these therapies show promise, their clinical efficacy remains under active investigation and high-quality trials are needed to validate their esffectiveness in modulating hemodynamic homeostasis disequilibrium.

## Conclusion

Hemodynamic homeostasis disequilibrium represents a critical transitional state in the host, characterized by systemic oxygen imbalance and the collapse of integrated cardiovascular-respiratory regulation. While directly involving the heart, vasculature, and lungs, this state also reflects broader pathophysiological disturbances encompassed by HOUR, including neural and autonomic failure, immune and coagulation dysfunction, bioenergetic and metabolic derangement, mitochondrial dysfunction.

## Take home message

Hemodynamic homeostasis disequilibrium represents a critical state of systemic oxygen imbalance, primarily driven by cardiovascular-respiratory dysfunction and closely linked to HOUR. Restoration of hemodynamic homeostasis requires integrated modulation of cardiovascular-respiratory coordination, neuroimmune responses, and cellular and mitochondrial function.
